# Resveratrol Attenuates Ischemia/Reperfusion Injury in Neonatal Cardiomyocytes and Its Underlying Mechanism

**DOI:** 10.1371/journal.pone.0051223

**Published:** 2012-12-20

**Authors:** Min Shen, Rui-Xin Wu, Lei Zhao, Juan Li, Hai-Tao Guo, Rong Fan, Yan Cui, Yue-Min Wang, Shu-Qiang Yue, Jian-Ming Pei

**Affiliations:** 1 Department of Physiology, National Key Discipline of Cell Biology, Fourth Military Medical University, Xi'an, People's Republic of China; 2 Department of Cardiology, Xijing Hospital, Fourth Military Medical University, Xi'an, People's Republic of China; 3 Department of Clinical Nursing, Fourth Military Medical University, Xi'an, People's Republic of China; 4 Department of Hepatobiliary and Pancreas Surgery, Xijing Hospital, Fourth Military Medical University, Xi'an, People's Republic of China; University of Western Ontario, Canada

## Abstract

This study was designed to investigate whether Resveratrol (Res) could be a prophylactic factor in the prevention of I/R injury and to shed light on its underlying mechanism. Primary culture of neonatal rat cardiomyocytes were randomly distributed into three groups: the normal group (cultured cardiomyocytes were in normal conditions), the I/R group (cultured cardiomyocytes were subjected to 2 h simulated ischemia followed by 4 h reperfusion), and the Res+I/R group (100 µmol/L Res was administered before cardiomyocytes were subjected to 2 h simulated ischemia followed by 4 h reperfusion). To test the extent of cardiomyocyte injury, several indices were detected including cell viability, LDH activity, Na^+^-K^+^-ATPase and Ca^2+^-ATPase activity. To test apoptotic cell death, caspase-3 activity and the expression of Bcl-2/Bax were detected. To explore the underlying mechanism, several inhibitors, intracellular calcium, SOD activity and MDA content were used to identify some key molecules involved. Res increased cell viability, Na^+^-K^+^-ATPase and Ca^2+^-ATPase activity, Bcl-2 expression, and SOD level. While LDH activity, capase-3 activity, Bax expression, intracellular calcium and MDA content were decreased by Res. And the effect of Res was blocked completely by either L-NAME (an eNOS inhibitor) or MB (a cGMP inhibitor), and partly by either DS (a PKC inhibitor) or Glybenclamide (a K_ATP_ inhibitor). Our results suggest that Res attenuates I/R injury in cardiomyocytes by preventing cell apoptosis, decreasing LDH release and increasing ATPase activity. NO, cGMP, PKC and K_ATP_ may play an important role in the protective role of Res. Moreover, Res enhances the capacity of anti-oxygen free radical and alleviates intracellular calcium overload in cardiomyocytes.

## Introduction

Acute myocardial infarction (AMI) is the most common type of cardiovascular diseases with a high mortality and morbidity nowadays. Fundamentally, AMI leads to the death of a great many of cardiomyocytes. Although the treatment has been improved a lot, the recovery of patients is still unsatisfactory. One of the most important factors responsible for the poor recovery is ischemia/reperfusion (I/R) injury occurring during the treatment [1], which leads more cardiomyocytes to death and weakens the effect of reperfusion therapy. I/R injury is a complicated process which involves various mechanisms. And apoptosis, as one of the most important mechanisms involved in I/R injury, plays an important role in the initiation and progression of I/R injury [2]. Kajstura et al [3] showed that apoptosis was the predominant mode of cardiomyocyte death induced by I/R. Also, there are some other factors [4], such as calcium overload, and reactive oxygen species (ROS) generation, contributing to cell death during I/R injury.

Apoptosis is positively and negatively regulated by the Bcl-2 protein family [5]–[7]. Proapoptotic proteins include Bax, Bak, Bcl-XS, Bad, Bid, Bik, Bim, Hrk, and Bok, whereas antiapoptotic proteins include Bcl-2, Bcl-XL, Bcl-w, Mcl-1, and A1/Bfl-1. Apoptosis is mediated by two pathways [8]——the extrinsic pathway and the intrinsic pathway [9]. The extrinsic pathway induces apoptosis through the binding of the ligands to the cell surface receptors, whereas the intrinsic pathway involves the mitochondria and endoplasmic reticulum (ER), and both of the pathways lead to caspase activation. Calcium overload triggers myocardial injury during I/R. An increase in mitochondrial Ca^2+^ concentration results in the depolarization of the inner mitochondrial membrane, the production of ROS, and the opening of the mitochondrial permeability transition pore (MPTP, a kind of nonspecific pore in the inner mitochondrial membrane permeable to small molecules). The opening of the MPTP induces either apoptosis or necrosis, which is the result of proapoptotic mitochondrial proteins release and the result of ATP depletion, respectively [10].

Resveratrol (Res) is a polyphenolic compound which mainly exists in red grapes and wine. In a previous epidemiological study [11] on the relationship between eating habits and coronary heart disease, an interesting phenomenon suggests that in all developed countries, the French consume the most quantity of wine on the average, but have the lowest morbidity of coronary heart disease. This phenomenon is called the “French paradox”, which has probably benefited from Res in wine. Res has extensive pharmacological effects, including anticancer [12]–[15], modulating inflammation response [16]–[18], improving ifosfamide-induced Fanconi syndrome in rats [19], treating diabetic nephropathy [20] and protecting neurons [21], [22]. Moreover, there have been additional studies implicating the cardioprotective role of Res [23]. However, the underlying mechanism involved in the protective role of Res is still not completely clear.

Therefore, the aims of the present study were to: (1) determine the mechanisms involved in the protective effect of Res. (2) ascertain several important molecules involved in Res' cardioprotective effect. (3) elucidate the prophylactic role of Res against I/R.

## Materials and Methods

### Reagents

Res was purchased from Sigma Chemical Co. (St. Louis, MO, USA). Fetal bovine serum and DMEM were purchased from Gibco Co. Trysin and Methyl thiazolyl tetrazolium (MTT) were purchased from Sigma Chemical Co. (St. Louis, MO, USA). DNA-Prep Stain and AnnexinV-FITC were purchased from Coulter Co. ApoAlert Caspase Colorometric Assay Kit was purchased from Clontech Co. ATPase assay kit and LDH assay kit were purchased from JianCheng Bioengineering Institute (Nanjing, China).

### Animals

Sprague-Dawley rats (1–3 days old) were purchased from the Center of Experimental Animal in the Fourth Military Medical University, China. All animals used in this study were cared for in accordance with the Guide for the Care and Use of Laboratory Animals published by the United States National Institute of Health (NIH publication no. 85-23, revised 1996), and all procedures were approved by the Committee of Experimental Animals of the Fourth Military Medical University.

### Primary culture of neonatal rat cardiomyocytes and I/R model

Primary cultures of neonatal rat cardiomyocytes cardiomyocytes from 1-to 3-day-old Sprague-Dawley rats were prepared by the method reported previously [24]. According to previous methods, the in vitro I/R model was employed in this study. Cardiomyocytes were initially perfused in normal Hank's solution with a gas mixture of 95% O_2_–5% CO_2_ at 37°C, pH 7.4. To simulate ischemia the Hank's solution was switched to pH 7.4 at 37°C without glucose or calcium (D-Hank's solution) and then the cells were aerated with a gas mixture of 95% N_2_–5% CO_2_. After that, to simulate reperfusion, the cells were again treated with normal Hank's solution with a gas mixture of 95% O_2_–5% CO_2_. In this way, in vitro I/R model was built.

### Experimental groups

1) Normal group: cardiomyocytes were incubated in normal Hank's solution during the entire experimental period; 2) I/R group: cardiomyocytes were treated with 2 h ischemia followed by 4 h reperfusion; 3) Res+I/R group: Res (100 µmol/L) was given 15 min before cardiomyocytes were subjected to I/R as described above in I/R group.

To explore several key signal molecules involved in the mechanism of Res' cardioprotective role, several blockers were used, and several new groups were formed as follows: 1) Normal group: cardiomyocytes were incubated in normal Hank's solution during the entire experimental period; 2) I/R group: cardiomyocytes were treated with 2 h ischemia followed by 4 h reperfusion; 3) Res+I/R group: Res (100 µmol/L) was given 15 min before cardiomyocytes were subjected to I/R; 4) L-NAME+Res+I/R group: L-NAME (1 mmol/L, a nitric oxide synthase inhibitor) and cardiomyocytes were incubated in Hank's solution for 30 min and Res was added. After 15 min, the group was subjected to I/R.; 5) MB+Res+I/R group: Methylene blue (MB at 50 µmol/L, a cGMP inhibitor) and cardiomyocytes were incubated in Hank's solution for 30 min and Res was added. After 15 min, the group was subjected to I/R; 6) DS+Res+I/R group: D-Sphingosine (DS at 0.1 µmol/L, a PKC inhibitor) and cardiomyocytes were incubated in Hank's solution for 30 min and Res was added. After 15 min, the group was subjected to I/R; 7) Gly+Res+I/R group: Glibenclamide (Gly at 10 µmol/L, a K_ATP_ inhibitor) and cardiomyocytes were incubated in Hank's solution for 30 min and Res was added. After 15 min, the group was subjected to I/R.

### Assay of cell viability

#### Assay of cell viability with trypan blue transmission method

When the experiment was over, the cell monolayer was stained with 2% trypan blue dissolved in HEPES-BSS for 5 min (37°C) and then was washed with HEPES-BSS for several times. Subsequently, 1.5% glutaraldehyde-0.1M sodium dimethy arsenate/HCl buffer solution was employed to fix it. Finally, we observed it with microscope to identify cell viability. When the cell membrane gets damaged, the cell is stained. So the dead cells were stained blue, while the viable ones were transparent. The cell viability was calculated according to the following formula:
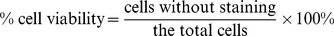



#### MTT colorimetry

The exogenous MTT can be reduced to insoluble purple crystal sediment, which dissolves in DMSO, within the cells by succinate dehydrogenase in the mitochondria of viable cells, rather than of dead cells. 20 µl MTT (5 mg/ml) was added to each well and the cells were incubated for 4 h till the end. After supernatant was abandoned, DMSO was added to dissolve the purple crystals within the cells. The absorbance of each well at 570 nm was measured with an enzyme-linked immune analyzer. Because the absorbance indicates the activity of succinate dehydrogenase within the mitochondria, the cell viability was calculated by the following formula:
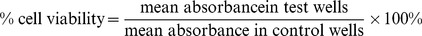



### Assay of lactate dehydrogenase (LDH) activity

The extent of cell injury was monitored by measuring LDH leakage. According to the manufacturer's instruction, 100 µL of culture medium was taken to assess LDH activity using a commercial kit with a spectrophotometer.

### Assay of Na^+^-K^+^-ATPase and Ca^2+^-ATPase

The extent of cellular injury was also monitored by Na^+^-K^+^-ATPase and Ca^2+^-ATPase activity. According to manufacturer's instruction, the kit was employed to detect the activity.

### TUNEL staining

Transferase dUTP nick end labelling (TUNEL) apoptotic analysis was performed using DeadEnd™ Fluorimetric TUNEL System (Promega, Madison, WI, USA) as described by the manufacturer.

### Annexin V-FITC/PI double marking flow cytometry

Fluorescein isothiocyanate (FITC)-conjugated Annexin V and propidium iodide (PI) (Annexin-V-FLUOS, Boehringer Mannheim, Mannheim, Germany) were used to identify apoptotic cells. In the figure of flow cytometry, the left lower quadrant indicates viable cells (Annexin V^−^/PI^−^); the right upper quadrant indicates necrotic cells (Annexin V^+^/PI^+^); the right lower quadrant indicates apoptotic cells(Annexin V^+^/PI^−^).

### Measurement of caspase-3 activity

Caspase-3 activity was evaluated by using a commercialized caspase-3 assay kit (Biovision, Inc.). Approximately 2×10^6^ cells were harvested by centrifugation, and the pellet was resuspended in lysis buffer. Protein levels were determined with the bicinchoninic acid assay (Beyotime Biotechnology, Shanghai, China). As described in the manufacturer's instructions, aliquots of protein (10 µL) were incubated with 10 µL of synthetic peptide substrate Ac-DEVD-pNA in a total volume of 100 µL at 37°C for 2 h to detect caspase-3 activity. Caspase-3 activity was expressed as optical density. The absorbance at 405 nm of the released pNA was monitored in a spectrophotometer. The relative activity of enzyme is monitored by the rate of absorption value in treatment group and normal group and we suppose the activity in normal group is 1.

### Detection of Bcl-2/Bax expression in cardiomyocytes with immunohistochemistry

The three groups of cardiomyocytes were fixed with 95% ethanol for 15 min. After inactivating endogenous peroxidase with 3% H_2_0_2_/PBS, and blocking cross-reactivity with normal serum for 30 min, the cells were incubated overnight at 4°C with the rabbit anti-Bcl-2 IgG (1∶100, Santa Cruz, California, USA) and the mouse anti-Bax IgG (1∶100, Santa Cruz, California, USA), followed by incubation with peroxidase-conjugated secondary anti-rabbit and anti-mouse antibodies respectively (1∶100) respectively. Then, the peroxidase was visualized with diaminobenzidine (DAB). The sections were dehydrated in graded ethanol, soaked in xylene, and mounted with Eukitt. The sections were examined with a light microscope. Cytomembrane and cytoplasm stained brown in Bax- and Bcl-2-positive cells. The expression of Bcl-2 and Bax was monitored by mean optical density.

### Assay of SOD activity and MDA content

The activity of SOD and MDA content were detected as described by the manufacturer.

### Measurement of intracellular calcium in different extracellular fluid

The laser scanning confocal microscope (LSCM) was used to measure the intracellular calcium according to the instructions described by the manufacturer and previous study [25]. The effect of H_2_O_2_ (100 µmol/L) on intracellular calcium (ECF is Hank's solution) was detected. The cells were incubated with Res (ECF is Hank's solution) for 10 min followed by H_2_O_2_ (100 µmol/L) and intracellular calcium was detected.

### Statistical Analysis

Data are expressed as means ± SD. Statistical analyses of data were performed by one-way ANOVA followed by the Student-Newman-Keuls test. A value of P<0.05 was considered to be statistically significant. All data analyses were conducted with the SPSS 13.0 software package (SPSS, Inc., Chicago, IL).

## Results

### Effect of Res on cell viability

**Figure 1:**
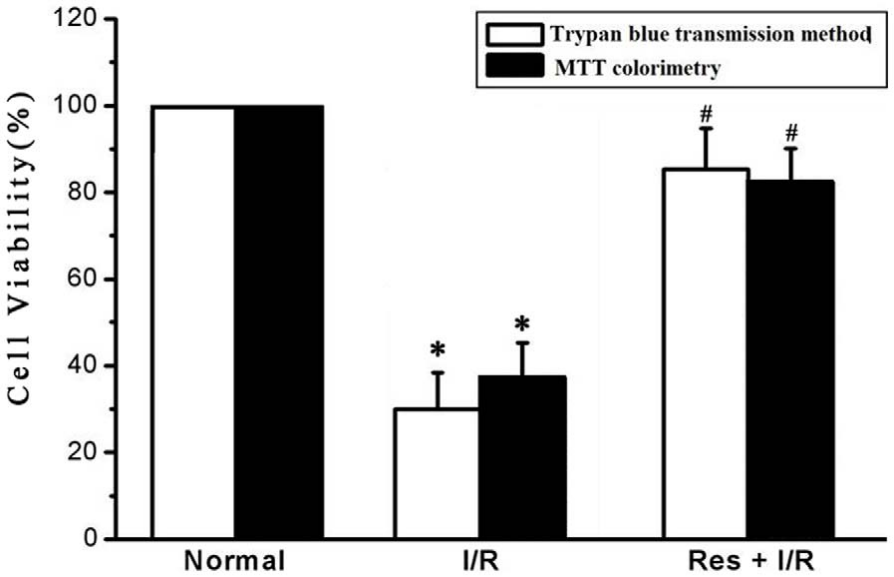
Effect of Res on cell viability by trypan blue method and methyl thiazolyl tetrazolium (MTT) colorimetry. The two methods revealed that Res stabilized cell membrane and increase cell viability during I/R. Res, resveratrol; I/R, ischemia/reperfusion. ^*^P<0.01 versus normal group, ^#^P<0.01 versus I/R group. Values are the means ± SD, n = 10 in each group.

By using trypan blue transmission method, the blue-stained cells in the I/R group increased and it was of significance compared with the normal group. Res+I/R group suggested dramatically increased transparent cells which indicated that Res stabilized cell membrane and increased cell viability during I/R. The result was of significance compared with the I/R group (Fig. 1).

In the experiment of MTT colorimetry, compared with optical density (OD) of cardiomyocytes in the I/R group, the cell viability in the Res+I/R group was much higher than that in the I/R group (Figure 1).

### Effect of Res on LDH, Na^+^-K^+^-ATPase and Ca^2+^-ATPase activity

In the I/R group, the membrane permeability increased, resulting in the elevation of LDH. In the Res+I/R group, the activity of LDH was significantly decreased (Table 1). The activity of Na^+^-K^+^-ATPase and Ca^2+^-ATPase in the I/R group was significantly lower than the normal group, which indicated that I/R attenuated Na^+^-K^+^-ATPase and Ca^2+^-ATPase function. Res elevated the activity of Na^+^-K^+^-ATPase and Ca^2+^-ATPase in the Res+I/R group compared with the I/R group, which suggested that Res protects activity of Na^+^-K^+^-ATPase and Ca^2+^-ATPase during I/R (Table 1).

**Table 1:** Effect of Res on LDH, Na^+^-K^+^-ATPase and Ca^2+^-ATPase.

Group	LDH (U/L)	Na^+^-K^+^-ATPase (µmol/mg/hour)	Ca^2+^ -ATPase (µmol/mg/hour)
Normal	658.1±97.2	0.572±0.141	0.751±0.123
I/R	2517.9±124.5**	0.478±0.088*	0.495±0.117**
Res+I/R	758.8±54.6##	0.545±0.112#	0.683±0.137##

* *P*<0.05,

** *P*<0.01 versus normal group.

# *P*<0.05,

## *P*<0.01 versus I/R group.

LDH: lactate dehydrogenase. Values are the means±SD, n = 10.

### Effect of Res on cardiomyocyte apoptosis

**Figure 2:**
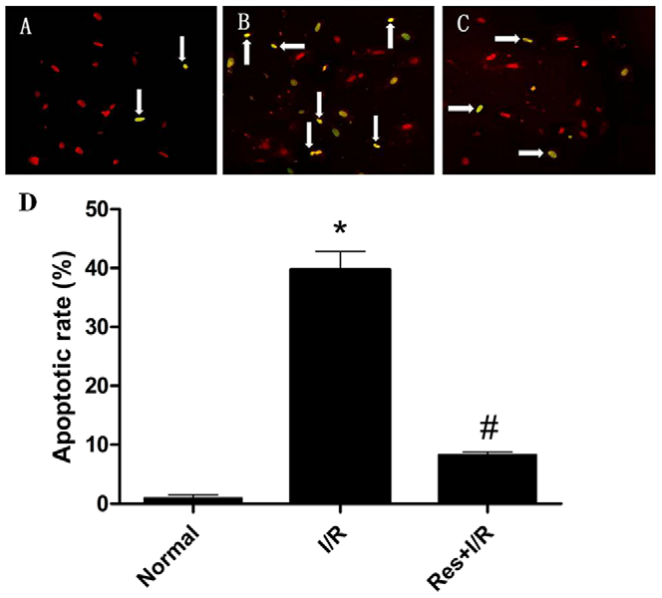
TUNEL staining and the apoptotic rate of cardiomyocytes. The arrow refers to apoptotic cells. (A) In the normal group, a very few apoptotic cells were found. (B) In the I/R group, the apoptotic cells increased dramatically. (C) In the Res+I/R group, the number of apoptotic cells was far less than that in the I/R group, which suggested that Res lowered cell apoptosis induced by I/R. (D) The statistical data of apoptotic rate. ^*^P<0.01 versus normal group, ^#^P<0.01 versus I/R group. Values are the means ± SD, n = 10 in each group.

As shown in Figure 2, under the laser confocal fluorescence microscopy, TUNEL staining showed that the apoptotic cells increased dramatically in the I/R group and Res lowered apoptotic cells obviously (Figure 2).

**Figure 3:**
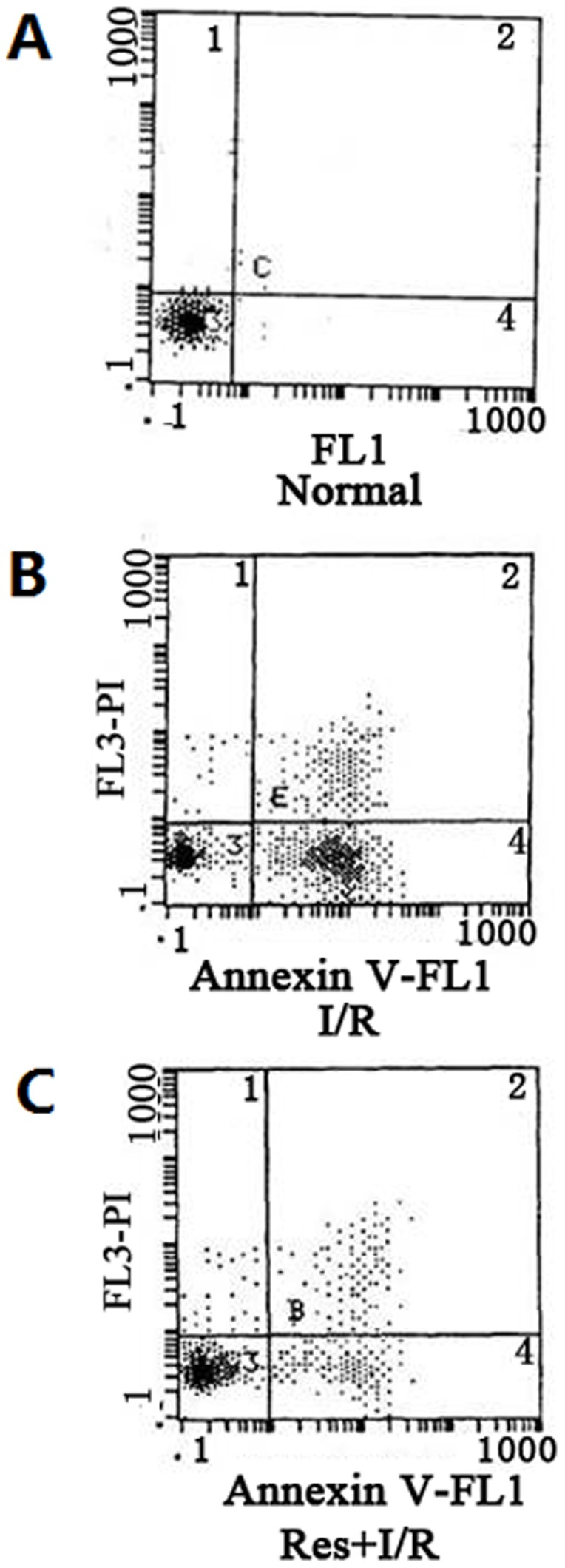
Assay of cardiomyocytic apoptosis with flow cytometry. (A) In the normal group, most of the cells lied in left lower quadrant, which indicated that the cells were viable. (B) In the I/R group, the number of cells in the right lower quadrant increased obviously compared with that in (A), which suggested that I/R injury induced apoptosis. (C) In the Res+I/R group, the number of cells in the right lower quadrant decreased dramatically compared with that in (B), which suggested that Res lowered cell apoptosis triggered by I/R.

A large number of Annexin V positive cardiomyocytes were detected in the I/R group, when the I/R group was pretreated with Res, Annexin V positive cardiomyocytes diminished distinctly (Figure 3). The apoptosis percentage is shown in table 2.

**Table 2:** Effect of Res on cardiomyocyte apoptosis percentage, the activity of caspase-3 and expressions of Bcl-2/Bax.

Group	cardiomyocyte apoptosis percentage (%)	the relative activity of caspase-3	Bcl-2 (optical density)	Bax (optical density)
Normal	0	1	138.9±8.4	101.4±4.2
I/R	39.7±5.4**	5.9±0.7**	99.5±4.8**	140.7±8.6**
Res+I/R	8.3±0.8##	2.8±0.4##	119.4±7.1##	125.3±5.8##

** *P*<0.01 versus normal group.

## *P*<0.01 versus I/R group. Values are the means±SD, n = 10.

### Effect of Res on the activity of caspase-3 and expressions of Bcl-2 and Bax

The cardiomyocytes in the I/R group showed elevated caspase-3 activity. When treated with Res, the activity of caspase-3 decreased dramatically (Table 2).

**Figure 4:**
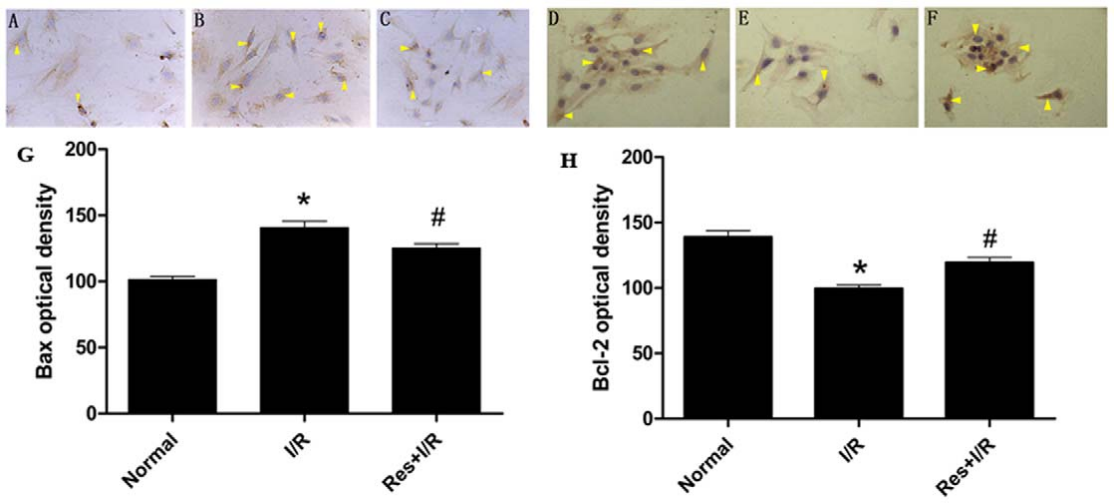
The expression of Bax and Bcl-2 detected by immunohistochemistry. The expression of Bax and Bcl-2 were shown and arrows refer to them. (A)(D)In normal condition, Bax and Bcl-2 were expressed in cardiomyocytes. (B)(E) I/R injury promoted Bax expression and suppressed Bcl-2 expression. (C)(F)On the contrary, Res suppressed Bax expression and promoted Bcl-2 expression. (G) The statistical data of Bax optical density. (H) The statistical data of Bcl-2 optical density. ^*^P<0.01 versus normal group, ^#^P<0.01 versus I/R group. Values are the means ± SD, n = 10 in each group.

In the normal condition, Bcl-2 can be expressed in cardiomyocytes. I/R injury suppressed Bcl-2 expression and promoted Bax expression. On the contrary, Res promoted Bcl-2 expression and suppressed Bax expression (Figure 4, Table 2).

### Effect of different blockers on the cardioprotective role of Res

The cell viability in normal group was 94.7%±6.1%. After cardiomyocytes were exposed to L-NAME, MB, DS and Gly for 1 hour, the cell viability were 95.0%±3.8%, 93.7%±5.3%, 96.1%±4.2% and 94.4%±4.6% respectively, which were not significantly different compared with the normal group(n = 10, P>0.05), indicating these drugs had no effect on the cell viability of cardiomyocytes under the normal conditions.

**Figure 5:**
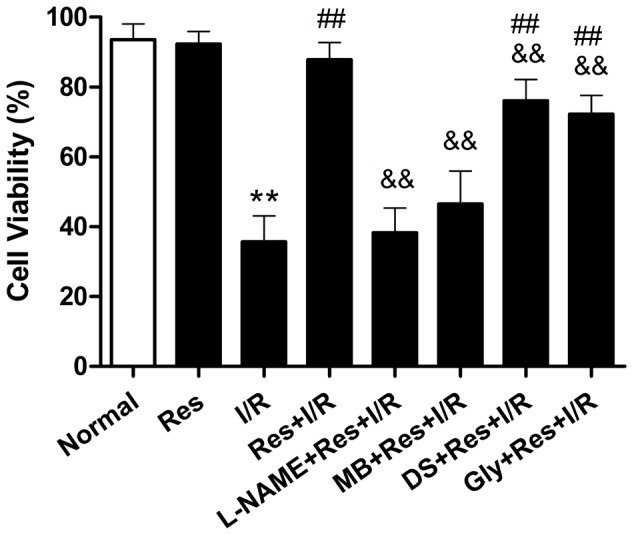
Effect of L-NAME, MB, DS and Gly on the cardiomyocytes viability in Res+I/R group. Res protected the cell viability against I/R, the role of Res on the cell viability was almost abolished by L-NAME and MB, and was significantly attenuated by DS and Gly. L-NAME, a nitric oxide synthase inhibitor; MB, Methylene blue, a cGMP inhibitor; DS, D-Sphingosine, a PKC inhibitor; Gly, Glibenclamide, a K_ATP_ blocker. ^**^ *P*<0.01 versus the normal group. ^##^ *P*<0.01 versus the I/R group. ^&&^ *P*<0.01 versus the Res+I/R group. Values are the means ± SD, n = 10 in each group.

The cell viability in the I/R group was 35.7%±7.4%, which was significantly reduced compared with the normal group. The cell viability in the Res+I/R group was 87.8%±4.9%, which had a significant difference compared with I/R group. After cardiomyocytes were exposed to L-NAME (an eNOS inhibitor), MB(a cGMP inhibitor), DS(a PKC inhibitor) and Gly(a K_ATP_ inhibitor) for 30 min and exposed to Res for 15 min, the cell viability after I/R injury were 38.3%±7.1%, 46.3%±9.7%, 76.2%±6.6% and 72.2%±5.4% respectively, of which the role of Res on the cell viability was almost abolished by L-NAME and MB, and was significantly attenuated by DS and Gly (Figure 5).

**Figure 6:**
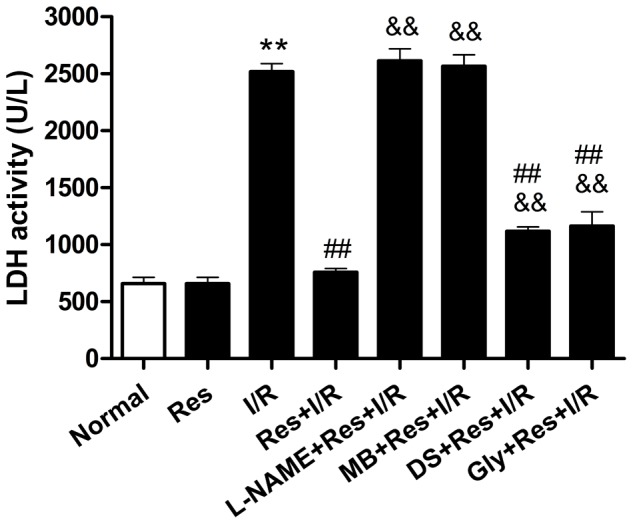
Effect of L-NAME, MB, DS and Gly on the LDH activity in Res+I/R group. The LDH activity in normal group was 658.1±97.2, while that in I/R group was 2517.9±124.5 and in Res+I/R group 758.8±54.6, which had a significant difference compared with I/R group. The effect of Res on the LDH activity was abolished by L-NAME and MB, and was significantly attenuated by DS and Gly. L-NAME, a nitric oxide synthase inhibitor; MB, Methylene blue, a cGMP inhibitor; DS, D-Sphingosine, a PKC inhibitor; Gly, Glibenclamide, a K_ATP_ blocker. ^**^ *P*<0.01 versus the normal group. ^##^ *P*<0.05 versus the I/R group. ^&&^ *P*<0.05 versus the Res+I/R group. Values are the means ± SD, n = 10 in each group.

The LDH activity in the normal group was 658.1±97.2 U/L, while that in the I/R group was 2517.9±124.5 U/L and in the Res+I/R group 758.8±54.6 U/L, which had a significant difference compared with the I/R group. The effect of Res on the LDH activity was abolished by L-NAME and MB, and was significantly attenuated by DS and Gly (2614.3±182.5 U/L, 2566.3±172.1 U/L, 1119.8±61.9 U/L, and 1162.8±216.5 U/L respectively) (Figure 6).

### 10.5

After I/R injury, SOD activity was elevated compared with normal cardiomyocytes, which was probably a protective reflex response. And the MDA content was also elevated significantly. The SOD activity in the Res+I/R group was elevated significantly compared with the I/R group, but MDA content was decreased significantly compared with the I/R group (Table 3).

**Table 3:** The effect of Res on SOD activity and MDA content in cardiomyocytes.

Group	SOD (U/ml)	MDA (µmol/L)
Normal	19.68±0.82	6.97±0.79
I/R	22.13±1.42*	13.55±1.69**
Res+I/R	29.28±1.24##	8.27±1.02##

* *P*<0.05,

** *P*<0.01 versus normal group.

## *P*<0.01 versus I/R group.

SOD = Superoxide Dismutase; MDA = Malondialdehyde. Values are the means±SD, n = 10.

### Effect of Res on intracellular calcium

**Figure 7:**
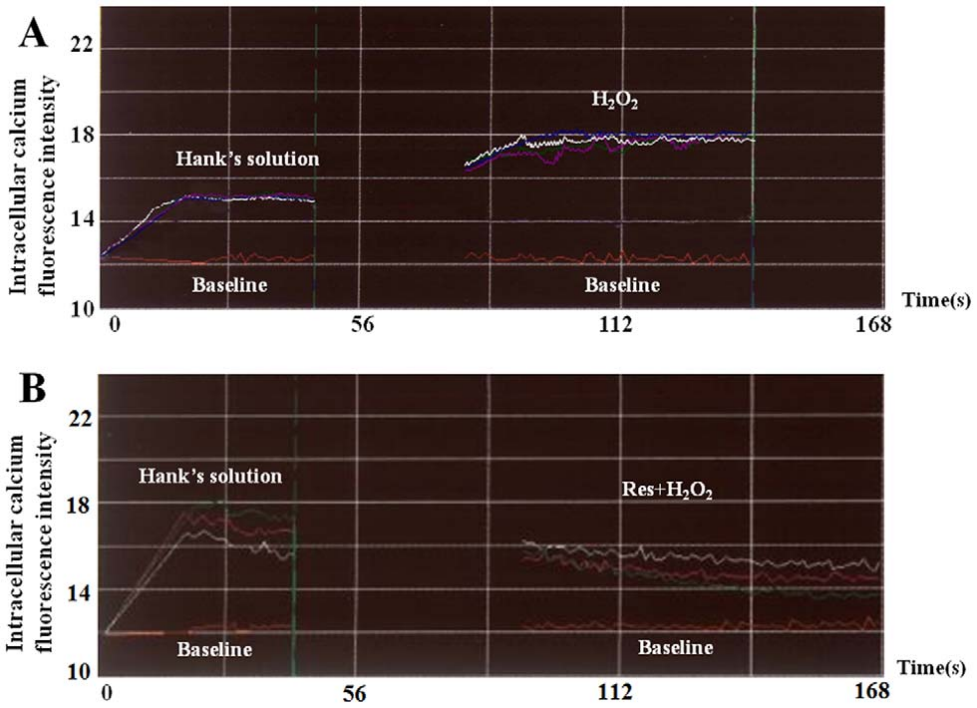
Effect of Res on the elevated intracellular calcium induced by H_2_O_2_. (A) In Hank's solution, H_2_O_2_ led to the increase of intracellular calcium, and the fluorescence intensity increased. (B) In Res group, the effect of H_2_O_2_ was abolished by Res.

In Hank's solution, intracellular calcium was elevated. H_2_O_2_ led to an increase of intracellular calcium, and the fluorescence intensity increased (Figure 7A). The effect of H_2_O_2_ was abolished by Res (Figure 7B).

## Discussion

The major findings in the present study are: (1) Res attenuates I/R injury in cardiomyocytes by preventing cell apoptosis decreasing LDH release and increasing ATPase activity. (2) NO, cGMP, PKC and K_ATP_ may play an important role in the protective effect of Res. (3) Res enhances the capacity of anti-oxygen free radical and alleviates intracellular calcium overload in cardiomyocytes.

Previous studies have suggested that Res has a cardioprotective effect [11], [23]. Our present study focused on the underlying mechanisms of the effect, suggesting that several pathways are involved in the process, such as anti-apoptosis and anti-oxygen free radical.

The fundamental result of myocardial ischemia lies in energy depletion. Ischemia may lead to free radical elevation, calcium overload and the destruction of cell membrane and mitochondria membrane, which contributes to energy depletion in the mitochondria. Those factors trigger the release of apoptosis induced factor (AIF) and Cytochrome C (Cyt C), activating cardiomyocytes apoptosis by mitochondrial pathway. Cell apoptosis of mitochondria pathway is mediated by Bcl-2 family proteins. It is acknowledged that *bcl-2* oncogene is the most important anti-apoptosis gene, and ischemia-induced apoptosis is associated with the low expression of *bcl-2*. The Bcl-2 family can be generally divided into two different groups——the anti-apoptotic members such as Bcl-2 and Bcl-xL, and the pro-apoptotic members such as Bax, Bak, Bid and Bad. In normal conditions, most of anti-apoptotic members in Bcl-2 family are isolated as membrane protein of organelle membrane and pro-apoptotic members are distributed in cytoplasm or cytoskeleton in inactivated forms. When triggered by stimuli, pro-apoptotic members have a conformational change and translocate into outer-membrane of mitochondria, resulting in the release of CytC and AIF which lead to apoptosis. It has been indicated that when Bcl-2∶Bax>1∶2, cell will survive and vice versa [26]. Bcl-2 is a pivotal molecule in many apoptosis pathways and it exerts inhibitory effect on free radical elevation, calcium overload and lipid peroxidation [27], [28]. It is generally acknowledged that Bcl-2 overexpression prevents AIF and Cyt C escaping from the mitochiondria and inhibits the activation of caspase-3, preventing the cardiomyocytes from apoptosis induced by ischemia and hypoxia [28].

It has been demonstrated that caspase plays a central role in the execution phase of apoptosis and are responsible for many of the morphological features normally associated with this form of cell death [29]. Caspase usually exists as proenzyme without activity, and only when a certain section of peptide in the proenzyme is cut off can it be actually activated. Caspase is a common pathway involved in apoptosis signal transduction. Activated by some pro-apoptotic factors, caspase then activates one another and consequently can initiate specific caspase cascades and apoptosis occur finally [30], [31].Caspase-3 is the way which must be passed through in the apoptosis cascade, and it is the key one in mammalian cell apoptosis [32]. In the present study, the activity of caspase-3 in cardiomyocytes was detected. The results indicated that I/R could elevate caspase-3 activity, but Res could decrease it dramatically. As the executioner of apoptosis, caspase-3 plays a pivotal role in apoptosis, and thus the protective role of Res is probably to be achieved by inhibiting caspase-3 activity.

In the present study, when cGMP inhibitor MB was added, the protective action of Res was totally blocked, which indicated that cGMP pathway plays an important role in the protective role of Res. It is widely acknowledged that PKC is an important protein kinase mediating ischemia preconditioning protective effect [33]. PKC activation and intracellular protein phosphorylation mediated by PKC are the keys to its protective action. However, the exact mechanism by which PKC provides myocardial functional protection is unclear. It is suggested that PKC may activate the cellular machinery required to prepare the heart to maintain homeostasis against I/R. K_ATP_ is also an important factor mediating ischemia preconditioning protective effect. It has been demonstrated that PKC activation promotes K_ATP_ opening, which shortens the action potential duration and thereby reduces ischemic Ca^2+^ overload [34], [35]; but some experiments found that when K_ATP_ agonist diazoxide served as a cardioprotective factor, it also activated PKC and led the translocation of PKC to mitochondria membrane. K_ATP_ channel blocker 5-hydroxydecanoate and PKC inhibitor Calphostin C could totally abolish the protective role of diazoxide in myocardial I/R. So, the relationship between K_ATP_ channel opening and PKC activation is very complicated and further research is needed to investigate that relationship. In a word, cGMP-dependent pathway is involved in the mechanism of Res' protective role. PKC activation and K_ATP_ opening are also involved in the mechanism.

Calcium overload is the common pathway involved in cell death. It has been demonstrated that the elevation in intracellular calcium triggers apoptosis; on the other hand, nifedipine (an L-type Ca^2+^ blocker) can block that effect [36]. An increase in mitochondrial Ca^2+^ concentration results in depolarization of the inner mitochondrial membrane, the production of reactive oxygen species, and the opening of the mitochondrial permeability transition pore (MPTP), which is a nonspecific pore in the inner mitochondrial membrane that is permeable to small molecules. Opening of the MPTP induces either apoptosis, as a result of the release of proapoptotic mitochondrial proteins, or necrosis, as a result of ATP depletion [37]. Therefore, the decrease of intracellular calcium can be a key factor in cardioprotection in I/R injury. In the present study, in Hank's solution environment, H_2_O_2_ led to an increase in intracellular calcium, while in Res group, the intracellular calcium was not elevated by H_2_O_2_. As injury induced by H_2_O_2_ simulation is produced by reoxygenation, Res resists calcium overload triggered by reoxygenation and serves as a cardioprotective factor.

I/R injury leads to oxygen free radical (OFR) generation and calcium overload, which are considered among the major factors of I/R injury. Hypoxia in cardiomyocytes triggers the depletion of ATP and the elevation of ADP and AMP. Then AMP was turned into hypoxanthine, which can lead to OFR generation in the condition of oxygen and xanthine oxidase. Xanthine dehydrogenase is rapidly turned into xanthine oxidase by calcium-dependent protease in hypoxia. When reoxygenation occurs in reperfusion, the OFR generation is promoted. OFR triggers lipid peroxidation, resulting in cell damage, which includes cell membrane permeability elevation, MDA generation and DNA damage. In the normal conditions, the OFR can be diminished by SOD,CAT and GSHps. However, the amount of OFR is beyond the capacity of those enzymes and cannot be diminished in reperfusion. Therefore, how to decrease the generation of OFR is the key to alleviating I/R injury. The duration of hypoxia is closely associated with I/R injury, so decreasing the hypoxia and ischemia duration is of great importance. Although reoxygenation triggers OFR generation, in fact the fundamental cause is still hypoxia and ischemia. So reperfusion is essential and necessary. The present study indicated that after I/R injury, SOD activity was elevated compared with the normal cardiomyocytes, which was probably a protective reflex response, and MDA content elevation, which is associated with lipid peroxidation.

In recent years, Sirt 1 has been shown to confer protection in various models of cardiovascular oxidative stress [38]–[40]. Sirt 1 is one of the family members of seven proteins termed sirtuins (SIRT 1-SIRT 7). This family has gained considerable attention for its impact on several important physiological processes associated with metabolism and stress resistance. Recent studies have suggested that SIRT1 is a key regulator of vascular endothelial homeostasis controlling angiogenesis, vascular tone and endothelial dysfunction.

On the one hand, SIRT1 was identified as a critical regulator of sprouting angiogenesis during vascular growth. Although endothelial cells expressed all sirtuin family members, knock down of SIRT1 was uniquely associated with a near total loss of sprouting angiogenesis in vitro [41]. On the other hand, SIRT1 plays a critical role in endothelial homeostasis by regulating the endothelial nitric oxide synthase (eNOS). Endothelial-derived nitric oxide (NO) regulates blood vessel relaxation and provides atheroprotective effects. Resveratrol, a polyphenolic activator of SIRT1, has been shown to increase the expression of eNOS [42] and the combination of resveratrol with the HMG-CoA reductase inhibitors (statins) increased the activation of eNOS resulting in increased functional recovery in a model of acute myocardial infarction [43]. Additionally, chronic resveratrol treatment improved endothelium-dependent relaxation in spontaneous hypertensive rats,however, it did not increase eNOS expression [44]. The role of SIRT 1 in the cardioprotective process of resveratrol remains controversial in the light of recent reports that resveratrol is not a direct SIRT 1 activator [45]. And the underlying mechanisms by which resveratrol enhance SIRT 1 activity remains poorly defined and needs further study [46].

In summary, Res contributes to cell survival in the following pathways: 1) it decreases intracellular calcium level so that apoptosis triggered by calcium overload is reduced; 2) it inhibits caspase-3 activity and leads to the high expression of Bcl-2 and the low expression of Bax, both of which are closely related with the mitochondria pathway of apoptosis; 3) it may produce cardioprotection via eNOS/NO and GC/cGMP pathways; 4) PKC and K_ATP_ channel are involved in mechanism; 5) it works as an anti-OFR agent by elevating SOD activity and decreasing MDA content; and there are still some other ways to be explored and explicated in the future.
